# Programming a serial killer: CAR T cells form non-classical immune synapses

**DOI:** 10.18632/oncoscience.406

**Published:** 2018-04-29

**Authors:** Alexander J Davenport, Misty R Jenkins

**Affiliations:** Immunology Division, Walter and Eliza Hall Institute of Medical Research, Parkville, VIC 3052, Australia

**Keywords:** Immune synapse, T cells, Chimeric Antigen Receptor, Cytotoxicity

Chimeric Antigen Receptors (CARs) are engineered immune receptors that underpin a promising new form of cellular immunotherapy for the treatment of cancer. Recently there have been two landmark FDA approvals for the use of Chimeric Antigen Receptor (CAR) T cell therapy for B cell malignancies, and with more than 300 CAR T cell clinical trials globally, it has become more important than ever to investigate the cell biology of these new “living” immunotherapies. The rapid pace at which these therapies have been translated, means that we still have a way to go in understanding how triggering T cells via a synthetic engineered receptor might alter the cell biology, function and persistence of CAR T cells. We have previously shown that CAR T cells have the ability to be serial killers, a property likely to be a key requirement for effective anti-tumour therapy and ultimately influencing the numbers of CAR T cells required for effective therapy [1].

Activation of cytotoxic T cells is initiated after formation of an immune synapse, a highly organized structure formed at the interface of the effector and target cell. The synapse is a dynamic structure where T cell signaling occurs, serine kinases are recruited and key effector proteins perforin and granzymes are secreted into, inducing target cell apoptosis. The synapse is comprised of a series of concentric rings or SupraMolecular Activating Clusters (SMACs) that have been likened to a “Bull's eye” structure. T cell receptor (TCR) signaling and termination occurs in the central SMAC, whilst the peripheral SMAC provides adhesion, and actin clears away to the distal SMAC [2].

We recently examined how triggering CAR T cells via CAR would alter the immune synapse. We utilized a dual-receptor transgenic mouse model, where the OTI TCR specific for SIINFEKL peptide and H-2Kb and a second generation anti-Her2 CAR (CD28-CD3ζ) were expressed by the same T cell [3]. We compared the formation of the immune synapse between either the TCR or the CAR in the same population of cells and showed that whilst TCR mediated responses facilitate the formation of a classical bull's eye structure, CAR T cell interactions were different [4]. The structure of the CAR immune synapse is a disorganized multifocal signaling cluster, as defined by Lck, which does not coalesce into a clearly defined structure. Interestingly, CAR T cells do not form a defined peripheral SMAC and unlike TCR- mediated interactions, CAR T cells don't rely on LFA-1 interactions to stabilize the immune synapse. In similar studies, a signaling molecule downstream of Lck, Zap70, has been reported to display a similar disorganized pattern in CD19-specific CAR T cells [5]. The strength of signal received by the T cell dictates its functional outcome. It is not yet known how differing CAR co-stimulatory domains and overall CAR design influences synapse formation or further downstream signaling. However, this disorganized feature of the CAR synapse held across affinities and species, as CAR T cells recognizing different antigens both display patchy signaling domains and actin clearance at the synapse [4].

In our current study, we demonstrate that faster proximal signaling occurs via CAR compared to TCR, which raises the possibility that high affinity CAR design could be further fine-tuned [4]. CAR T cells also display faster recruitment of lysosomes to the immune synapse indicating that were able to mount a more rapid killer response as compared to TCR triggering. Signal strength is influenced by the binding affinity for antigen, the avidity of interaction, and the duration of synapse dwell time resulting in a graded response to TCR signalling [6]. We have previously shown that T cell-target synapse dwell time correlates with cytokine and chemokine production [7] and that CAR T cells display a similar off-rate as compared to TCR interactions [1]. Previous studies have shown that lower affinity CAR T cells displayed more efficient tumour clearance [8], and how the correlation between CAR T cell synapse off-rate and affinity, relating to function is an area of our interest.

Taken together, our recent study highlights the need to further understand the mechanisms by which CAR T cells kill their target cells which will also provide insight to increase efficiency. Whilst CAR T cells have become a highly effective form of therapy in some haematological cancer, therapeutic application to solid tumours presents additional challenges. As CAR T therapy matures, further clinical investigations are underway combining CAR T therapy with checkpoint inhibition via anti-PD-1/CTLA- 4 and other chemotherapeutic drugs. Current CAR T cell clinical trials utilise a variety of CAR designs, therefore varying affinity and CAR design may result in different signalling thresholds and functional outcomes. As such CAR T cell functional ability will be influenced by their fundamental molecular design.
